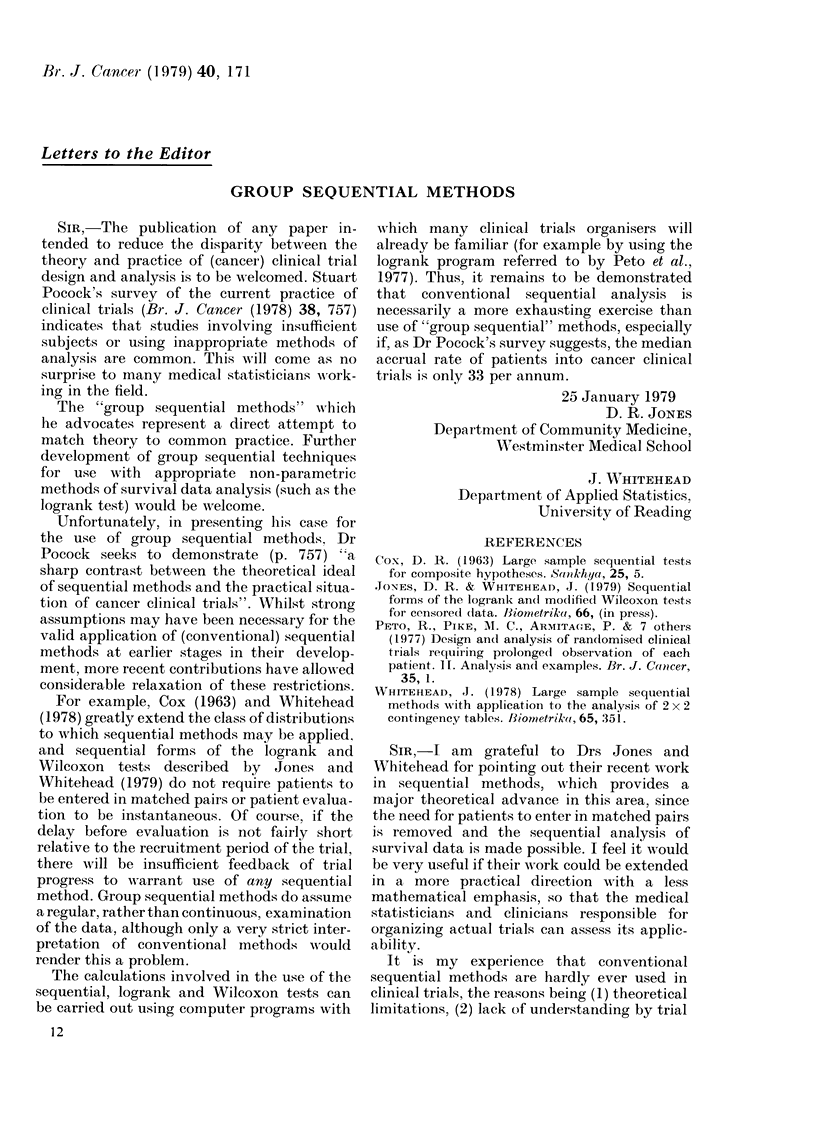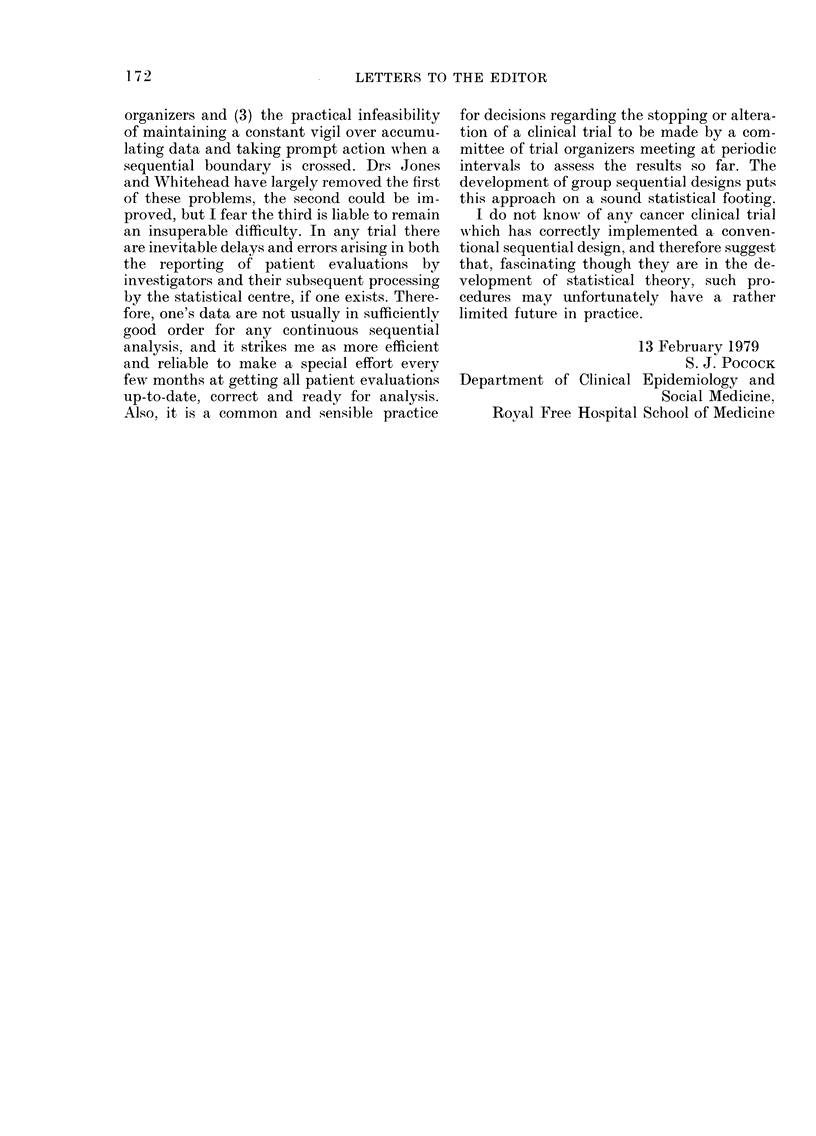# Group sequential methods.

**DOI:** 10.1038/bjc.1979.156

**Published:** 1979-07

**Authors:** D. R. Jones, J. Whitehead


*Br. J. Cancer* (1979) **40**, 171

### *Letters to the Editor*

### GROUP SEQUENTIAL METHODS

SIR,—The publication of any paper intended to reduce the disparity between the theory and practice of (cancer) clinical trial design and analysis is to be welcomed. Stuart Pocock's survey of the current practice of clinical trials (*Br. J. Cancer* (1978) **38, 757**) indicates that studies involving insufficient subjects or using inappropriate methods of analysis are common. This will come as no surprise to many medical statisticians working in the field.

The "group sequential methods" which he advocates represent a direct attempt to match theory to common practice. Further development of group sequential techniques for use with appropriate non-parametric methods of survival data analysis (such as the logrank test) would be welcome.

Unfortunately, in presenting his case for the use of group sequential methods, Dr Pocock seeks to demonstrate (p. 757) "a sharp contrast between the theoretical ideal of sequential methods and the practical situation of cancer clinical trials". Whilst strong assumptions may have been necessary for the valid application of (conventional) sequential methods at earlier stages in their development, more recent contributions have allowed considerable relaxation of these restrictions.

For example, Cox (1963) and Whitehead (1978) greatly extend the class of distributions to which sequential methods may be applied, and sequential forms of the logrank and Wilcoxon tests described by Jones and Whitehead (1979) do not require patients to be entered in matched pairs or patient evaluation to be instantaneous. Of course, if the delay before evaluation is not fairly short relative to the recruitment period of the trial, there will be insufficient feedback of trial progress to warrant use of *any* sequential method. Group sequential methods do assume a regular, rather than continuous, examination of the data, although only a very strict interpretation of conventional methods would render this a problem.

The calculations involved in the use of the sequential, logrank and Wilcoxon tests can be carried out using computer programs with which many clinical trials organisers will already be familiar (for example by using the logrank program referred to by Peto *et al.*, 1977). Thus, it remains to be demonstrated that conventional sequential analysis is necessarily a more exhausting exercise than use of "group sequential" methods, especially if, as Dr Pocock's survey suggests, the median accrual rate of patients into cancer clinical trials is only 33 per annum.

25 January 1979

D. R. JONES
Department of Community Medicine,
Westminster Medical School

J. WHITEHEAD
Department of Applied Statistics,
University of Reading

REFERENCES

COX, D. R. (1963) Large sample sequential tests for composite hypotheses. *Sankhya*, **25**, 5.

JONES, D. R. & WHITEHEAD, J. (1979) Sequential forms of the logrank and modified Wilcoxon tests for censored data. *Biometrika*, **66**, (in press).

PETO, R., PIKE, M. C., ARMITAGE, P. & 7 others (1977) Design and analysis of randomised clinical trials requiring prolonged observation of each patient. II. Analysis and examples. *Br. J. Cancer*, **35**, 1.

WHITEHEAD, J. (1978) Large sample sequential methods with application to the analysis of $2 \times 2$ contingency tables. *Biometrika*, **65**, 351.

SIR,—I am grateful to Drs Jones and Whitehead for pointing out their recent work in sequential methods, which provides a major theoretical advance in this area, since the need for patients to enter in matched pairs is removed and the sequential analysis of survival data is made possible. I feel it would be very useful if their work could be extended in a more practical direction with a less mathematical emphasis, so that the medical statisticians and clinicians responsible for organizing actual trials can assess its applicability.

It is my experience that conventional sequential methods are hardly ever used in clinical trials, the reasons being (1) theoretical limitations, (2) lack of understanding by trial organizers and (3) the practical infeasibility of maintaining a constant vigil over accumulating data and taking prompt action when a sequential boundary is crossed. Drs Jones and Whitehead have largely removed the first of these problems, the second could be improved, but I fear the third is liable to remain an insuperable difficulty. In any trial there are inevitable delays and errors arising in both the reporting of patient evaluations by investigators and their subsequent processing by the statistical centre, if one exists. Therefore, one's data are not usually in sufficiently good order for any continuous sequential analysis, and it strikes me as more efficient and reliable to make a special effort every few months at getting all patient evaluations up-to-date, correct and ready for analysis. Also, it is a common and sensible practice for decisions regarding the stopping or alteration of a clinical trial to be made by a committee of trial organizers meeting at periodic intervals to assess the results so far. The development of group sequential designs puts this approach on a sound statistical footing.

I do not know of any cancer clinical trial which has correctly implemented a conventional sequential design, and therefore suggest that, fascinating though they are in the development of statistical theory, such procedures may unfortunately have a rather limited future in practice.

13 February 1979

S. J. Pocock
Department of Clinical Epidemiology and Social Medicine,
Royal Free Hospital School of Medicine